# Volumineux kyste épidermoïde cervical compressif: à propos d’un cas clinique

**DOI:** 10.11604/pamj.2019.34.169.19925

**Published:** 2019-11-29

**Authors:** Nogognan Ignace Lengane, Souleymane Ouattara, Noé Zaghre, Milckisédek Judicaël Marouruana Some, Therese Ouedraogo, Bertin Priva Ouedraogo

**Affiliations:** 1 Service d’Oto-Rhino-Laryngologie et de Chirurgie Cervico-faciale, Centre Hospitalier Universitaire Régional de Ouahigouya, Ouahigouya, Burkina Faso; 2 Service de Laboratoire, Section Anatomo-pathologie, Centre Hospitalier Universitaire de Tingandogo, Tingandogo, Burkina Faso; 3 Service d’Oto-Rhino-Laryngologie et de Chirurgie Cervico-faciale, Centre Hospitalier Universitaire de Tingandogo, Tingandogo, Burkina Faso; 4 Service d’Imagerie Médicale et Radiodiagnostic, Centre Hospitalier Universitaire Régional de Ouahigouya, Ouahigouya, Burkina Faso

**Keywords:** Kyste épidermoïde, cervical, volumineux, Epidermoid cyst, cervical, voluminous

## Abstract

Le kyste épidermoïde est une tumeur bénigne d'origine embryonnaire. Il est lié à une localisation anormale du tissu ectodermique. La localisation cervicale est rare. Il peut poser des difficultés diagnostiques, surtout dans les formes volumineuses et compressives. Nous rapportons un cas de volumineux kyste épidermoïde du cou avec des signes de compression des voies aérodigestives supérieures. Le patient a bénéficié d'une cervicotomie avec exérèse de la masse. L'examen anatomopathologique de la pièce opératoire retrouvait un kyste épidermique.

## Abstract (en)

Epidermoid cyst is a benign embryonic tumor. It is an anomalous localization of ectoderm-derived tissue. Cervical epidermoid cyst is rare. It can cause diagnostic difficulties, especially in patients with voluminous and compressive cysts. We report a case of large epidermoid cyst of the neck with signs of compression of the upper aerodigestive tract. The patient underwent cervicotomy with mass resection. Pathological examination of the surgical specimen showed epidermal cyst.

## Introduction

Le kyste épidermoïde est une tumeur bénigne d'origine embryonnaire. Il est lié à une localisation anormale du tissu ectodermique. La paroi kystique est constituée par un épithélium malpighien [1-3]. Il peut être situé partout sur le corps avec une atteinte préférentielle des ovaires et des testicules [4]. La localisation cervicale est rare. Nous rapportons un cas de volumineux kyste épidermoïde du cou avec des signes de compression des voies aérodigestives supérieures.

## Patient et observation

**Figure 1:**
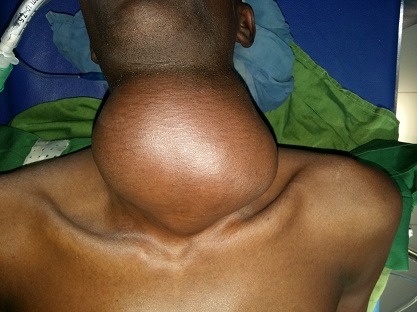
Masse cervicale antérieure

**Figure 2:**
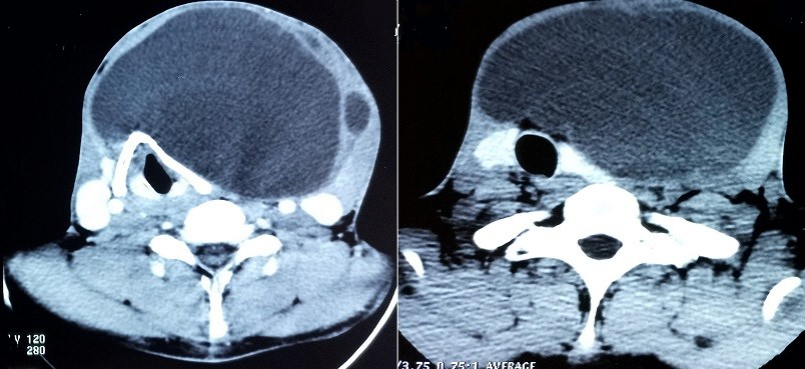
Coupes scannographiques axiales

**Figure 3:**
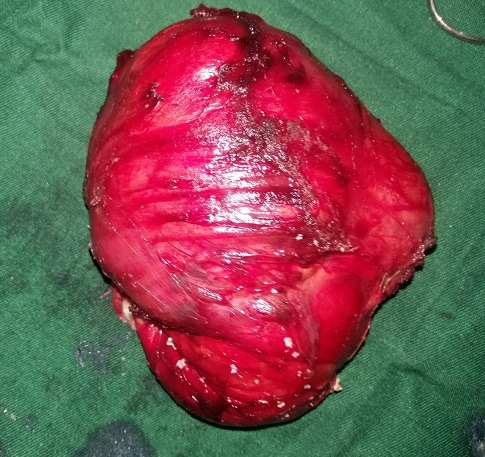
Pièce opératoire

**Figure 4:**
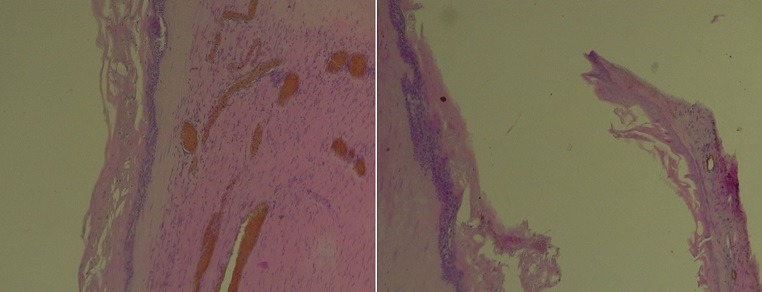
Prélèvement histologique coloré à l’hématéine-éosine (grossissement 40 et 100) montrant une paroi de kyste avec un revêtement malpighien kératinisant

Un patient de 32 ans a consulté au Service d'Oto-Rhino-Laryngologie (ORL) et Chirurgie Cervico-faciale pour une tuméfaction cervicale antérieure évoluant depuis 20 ans, associée à une dysphonie. L'examen clinique mettait en évidence une tuméfaction cervicale antérieure kystique, indolore, mobile (Figure 1). La tomodensitométrie objectivait une formation kystique multicloisonnée, bien limitée de 128x115mm, avec un rehaussement des cloisons après injections de produit de contraste. Elle refoule la thyroïde et les voies aérodigestives (Figure 2). Le patient a bénéficié d'une cervicotomie avec exérèse de la masse (Figure 3). Les suites opératoires ont été simples avec la sortie du patient 48h après l'intervention chirurgicale. L'examen anatomopathologique de la pièce opératoire retrouvait un kyste épidermique (Figure 4). Le suivi à 12 mois n'a pas noté de récidive.

## Discussion

Le kyste épidermoïde est un des trois kystes congénitaux de l'embryogenèse. Les deux autres étant le kyste dermoïde et le kyste teratoïde. Durant la fermeture du premier et du second arc branchial, l'inclusion de restes épithéliaux conduit à la formation de kystes épidermoïdes [3, 4]. Les kystes épidermoïde et dermoïde sont rares au niveau cervico-faciale. Ils représentent 7% des masses kystiques de la région, desquelles 1,6% surviennent dans la cavité buccale [1, 2, 5]. Le kyste épidermoïde peut être congénital ou acquis (post traumatique), sans qu'il n'y ait de différence sur le plan clinique ou histologique entre les deux. Il survient à tout âge, mais devient visible en général entre 15 et 35 ans avec une prédominance masculine [3, 5].

Sur le plan clinique, il s'agit des masses kystiques de la ligne médiane. Elles sont généralement asymptomatiques. Les signes fonctionnels sont liés au volume et à la localisation de la masse, surtout à proximité des structures vitales [6]. Il peut s'agir de dysphagie, de dysphonie et de dyspnée [3]. Il peut poser des difficultés diagnostiques dans la localisation cervicale, surtout dans les formes volumineuses et compressives comme chez notre patient. Le diagnostic différentiel des masses cervicales antérieures comporte, les masses thyroïdiennes, les kystes du tractus thyréoglosse, les kystes dermoïdes, les lymphangiomes kystiques [5-7]. A l'échographie, le kyste épidermoïde est bien limité avec une paroi épaisse avec des débris échogènes. Au scanner il apparait hypodense. A l'imagerie par résonnance magnétique, il apparait hypointense en T1 et hyperintense en T2 [2].

Sur le plan histologique, la paroi du kyste épidermoïde est constituée par un épithélium malpighien pluristratifié, kératinisant et le contenu de la cavité est composé par des squames de kératine [1, 5]. Le kyste dermoïde comporte un épithélium malpighien, pluristratifié, kératinisant et renfermant des annexes de la peau (glandes sudoripares, glandes sébacées) et des phanères (follicules pileux) [1, 5]. Le kyste teratoïde comporte en plus des éléments du kyste dermoïde, des éléments d'origine mésodermique (muscle, os, dents) [1, 8]. Le traitement repose sur l'exérèse chirurgicale complète. Les récidives sont rares [2]. Des cas isolés de lésions précancéreuse et cancéreuse ont été identifiés dans les parois kystiques, d'où la nécessité d'une analyse histologique de toute masse kystique [8, 9].

## Conclusion

Le kyste épidermoïde cervical est rare. Il peut poser des difficultés diagnostiques surtout dans les formes volumineuses et compressives. L'imagerie est d'un apport capital dans la démarche diagnostique et dans la planification de la chirurgie.

## Conflits d’intérêts

Les auteurs ne déclarent aucun conflit d'intérêts.
